# Contributions of avoidable mortality to the sex gap in life expectancy and life disparity in Iran

**DOI:** 10.1186/s13690-023-01141-z

**Published:** 2023-07-07

**Authors:** Mohsen Bayati, Ali Kiadaliri

**Affiliations:** 1 Health Human Resources Research Center, School of Management and Information Sciences, Shiraz University of Medical Sciences, Shiraz, Iran; 2 Clinical Epidemiology Unit, Department of Clinical Sciences Lund, Orthopaedics, Skåne University Hospital, Lund University, Remissgatan 4, Lund, SE-221 85 Sweden; 3 Centre for Economic Demography, Lund University, Lund, Sweden

**Keywords:** Life expectancy, Life disparity, Sex gap, Avoidable death, Decomposition

## Abstract

**Background:**

Public health policies and healthcare quality play a pivotal role on the health outcome level and disparities across sociodemographic groups. However, there is little evidence on their role on disparities in life expectancy (LE) and life disparity (LD) in low and middle income countries. The present study aimed to assess the contributions of avoidable mortality, as a measure of inter-sectoral public health policies and healthcare quality, into the sex gap in LE (SGLE) and LD (SGLD) in Iran.

**Methods:**

Latest available data of death causes, according to the ICD codes, for Iran was obtained from the WHO mortality database for the period 2015–2016. An upper age limit of 75 years was applied to define avoidable causes of death. LD was measured as the average years of life lost at birth. The SGLE and SGLD (both females minus males) were decomposed by age and cause of death using a continuous-change model.

**Results:**

Females, on average, outlived males for 3.8 years (80.0 vs. 76.2 years) with 1.9 lower life years lost (12.6 vs. 14.4 years). Avoidable causes accounted for 2.5 (67%) and 1.5 (79%) years of the SGLE and SGLD, respectively. Among avoidable causes, injury-related deaths followed by ischaemic heart disease had the greatest contributions to both SGLE and SGLD. Across age groups, the age groups 55–59 and 60–64 accounted for the greatest contributions of avoidable causes to SGLE (0.3 years each), while age groups 20–24 and 55–59 had the greatest contributions to SGLD (0.15 years each). Lower mortality rates for females than males in age groups 50–74 years accounted for about half of the SGLE, while age groups 20–29 and 50–64 years accounted for around half of SGLD.

**Conclusion:**

More than two third of the SGLE and SGLD in Iran were attributed to the avoidable mortality, particularly preventable causes. Our results suggest the need for public health policies targeting injuries in young males as well as lifestyle risk factors including smoking in middle aged males in Iran.

**Supplementary Information:**

The online version contains supplementary material available at 10.1186/s13690-023-01141-z.

**Table:** 

**Text box 1. Contributions to the literature**
• There is limited evidence on contributions of healthcare systems and public health policies in sex gaps in life expectancy (LE) and life disparity (LD), especially across developing countries.
• Avoidable causes of death, particularly injury, ischaemic heart disease and lung cancer account for majority of LE and LD disadvantages among Iranian males versus females.
• Public health policies targeting behavioral risk factors among younger Iranian males are urgently needed.

## Introduction

Life expectancy (LE), a primary measure of population health, is frequently used to evaluate health system performance [1]. LE has more than doubled globally over the past century. Similar to other countries, Iran also has experienced significant improvements in LE over recent decades [2]. While LE reflect the average length of life, it doesn’t provide information on variability in length of life. Indeed, LE can mask substantial variations in life span across subgroups in a population. Hence, to capture a comprehensive picture of a population health, LE should be complemented with lifespan inequality which quantifies the disparity in age at death in a population [3]. Different measures of lifespan inequality have been used in the literature including life disparity (LD) defined as the mean number of years lost due to death [4]. The relationship between LE and LD varies by a country’s LE level and stage of demographic transition [5]. In countries with low levels of LE, remarkable reduction in LD is correlated with increase in LE. On the other hand, in countries with high levels of LE, there isn’t large correlation between LD and rise in LE, and LD doesn’t decrease uniformly across all ages [4].

Although improving the overall health is a main objective of healthcare systems, ensuring an equitable distribution of health outcomes across different subgroups is also a crucial objective of healthcare systems. In this regard, health inequalities by sex/gender has attracted a lot of attention since these inequalities reflect differences in social structures including political and economic institutions and health and social policies [6]. Remarkable differences in LE between females and males is well-documented with females outliving males in all countries except Qatar and Afghanistan, by an average of 5.1 years globally in 2019 [7]. The sex gap in LE (SGLE) can be explained by biological, behavioral and socioeconomic factors [8, 9]. Moreover, given crucial role of health systems in improving population health and longevity through ensuring timely access to quality and affordable healthcare (both preventive and curative) [10, 11], it is very likely that potential sex gap in benefits of public health policies and quality healthcare can lead to the SGLE [12, 13]. Therefore, an important policy question is the extent to which health policy and healthcare quality contribute to the population health and distribution of health outcome between sociodemographic groups including males and females.

Avoidable death, defined as the unnecessary premature deaths that can be prevented by suitable and timely healthcare interventions and effective public health policies, has long been used as a measure of health system performance [14]. Avoidable mortality is generally divided into two main categories: preventable and treatable mortality. Preventable mortality is defined as the deaths that can be prevented by appropriate public health programs or primary health care interventions. Treatable or amenable mortality refers to the deaths that can be avoided with effective and timely medical interventions at secondary and tertiary healthcare levels [14]. Despite the importance of the issue, there is little research on the avoidable mortality and its role in the LE and LD in low and middle income countries including Iran. Previous studies in Iran were mainly conducted at the provincial level describing the proportions of avoidable deaths [15, 16]. An exception is a recent study investigating the contributions of avoidable causes to the gaps in LE and lifespan inequality between Iran and three neighbor countries [17]. However, the contribution of avoidable deaths to SGLE and SGLD is largely unexplored. The current study aimed to fill this knowledge gap by quantifying the contributions of avoidable deaths by cause and age to the SGLE and SGLD in Iran.

## Methods

This was a cross-sectional ecological study conducted in Iran. Latest available causes of death data for Iran was collected from the World Health Organization (WHO) mortality database for the years 2015–2016 (http://www.who.int/healthinfo/mortality_data/). The WHO Mortality Database provides annual data on causes of death by country, year, sex and age reported by member countries from their civil vital registration system. Causes of death data reported according to the International Classification of Diseases (ICD) codes. The underlying causes of death data was extracted by sex and age groups (< 1, 1–4, 5–9, …, > 85 years). Two-year period (2015–2016) death data was aggregated to avoid yearly random fluctuation. It should be noted that data for Iran were only available for period 2013–2016 and we selected the most recent period.

We identified avoidable causes of death using the list developed by the Organization for Economic Co-operation and Development (OECD) and the statistical office of the European Union (Eurostat) [18]. Accordingly, we categorized avoidable deaths into four mutually exclusive groups: 1) “only preventable”, “only treatable (amenable)”, “treatable and preventable”, and “ischemic heart disease (IHD)”. While IHD belongs to “treatable and preventable” category, we analyzed it as a separate group due to the large number of IHD deaths which can mask the contributions of other causes in this category. In line with previous studies, an upper age-limit was set at 75 years to define avoidable causes of death. This means that deaths in people aged 75 and older were considered as non-avoidable.

Sex-specific LE and LD were estimated using abridged life tables. LD (e^†^, “e-dagger”) quantifies the average remaining life expectancy at death (or alternatively the average years of life lost due to death) [4, 19]. Both SGLE and SGLD were computed as the difference between females and males. For interpretation, a positive (negative) SGLE reflects LE advantage in females (males) while a positive (negative) SGLD reflects LD advantage in males (females). The SGLE and SGLD were decomposed by age and cause of death using a continuous-change model suggested by Horiuchi et al. [20] and implemented in R program using the codes from the following open source: https://github.com/jmaburto.

## Results

During 2015–2016, LE was 80.0 years for females and 76.2 years for males. This corresponds to a SGLE of 3.8 years. On the other hand, LD was 12.6 years for females and 14.4 years for males, suggesting 1.9 years greater average life years lost for males than females.

### Cause-specific contributions to the sex gap in LE and LD

Avoidable causes were responsible for about 2.5 (67%) out of 3.8 years of the SGLE and 1.5 (79%) out of 1.9 years of the SGLD. Preventable causes mainly through injury-related deaths were the leading contributor of both SGLE and SGLD (Table 1). Indeed, the total contributions of treatable, IHD, and treatable & preventable causes (0.9 years to the SGLE and 0.4 years to the SGLD) were smaller than the contribution of injury-related death (1.1 and 0.8 years to the SGLE and SGLD, respectively). Following injury, IHD (0.6 and 0.3 years to the SGLE and SGLD, respectively) and preventable cancers including lung cancer (0.3 and 0.1 years to the SGLE and SGLD, respectively) had the greatest contributions to the SGLE and SGLD. While most causes contributed to LE/LD advantage among females over males, the opposite was seen for treatable cancers (e.g. breast cancer) and diabetes mellitus.

**Table 1:** Cause-specific contributions to the sex gap in life expectancy (SGLE) and life disparity (SGLD) in Iran over 2015–2016

Causes			SGLE	SGLD
Avoidable	*Ischemic heart disease (IHD)*		*0.605*	*-0.292*
	*Treatable & preventable*	Cerebrovascular diseases	0.105	-0.053
		Diabetes mellitus	-0.021	0.003
		Hypertensive diseases	0.060	-0.033
		Other causes	0.030	-0.015
		*Total*	*0.173*	*-0.098*
	*Preventable*	Alcohol-related deaths	0.030	-0.015
		Cancers (including lung cancer)	0.304	-0.120
		Drug-related deaths	0.083	-0.056
		Infectious diseases	0.051	-0.028
		Injuries	1.128	-0.774
		Other causes	-0.001	0.000
		Diseases of the respiratory system	0.070	-0.028
		*Total*	*1.666*	*-1.021*
	*Treatable*	Cancer	-0.171	0.091
		Diseases of the digestive system	0.012	-0.006
		Diseases of the genitourinary system	0.052	-0.029
		Infectious diseases	0.010	-0.006
		Pregnancy, childbirth and perinatal period	0.104	-0.068
		Other causes	0.012	-0.010
		Diseases of the respiratory system	0.065	-0.031
		*Total*	*0.083*	*-0.059*
	Total avoidable		2.527	-1.470
Non-avoidable			1.268	-0.398
All			3.795	-1.869

### Age-specific contributions to the sex gap in LE and LD

**Fig. 1:**
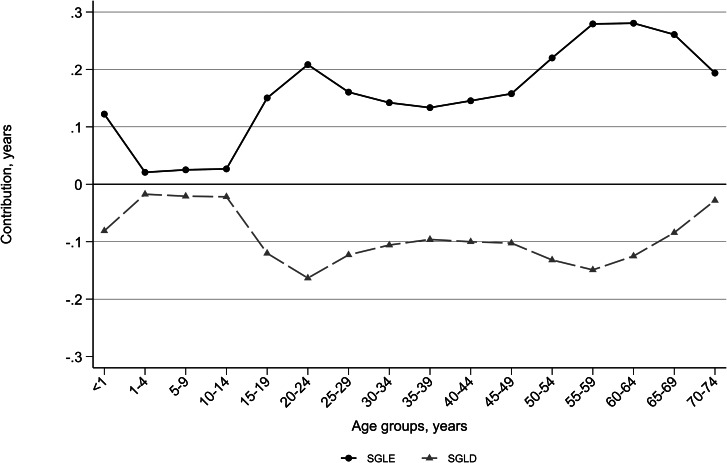
Age-specific contributions of avoidable deaths to the sex gap life expectancy (SGLE) and lifespan disparity (SGLD) in Iran over 2015–2016

Across all age groups, the age-specific mortality rates for avoidable causes were lower in females than males, translating into both LE and LD advantages for females over males (Fig. 1). In particular, lower mortality rates for females than males in age groups 50–74 years accounted for about half (1.2 out of 2.5 years) of the SGLE attributable to avoidable causes of death. On the other hand, age groups 15–29 and 50–64 years accounted for about 55% (0.8 out of 1.5 years) of the SGLD attributable to avoidable causes. The age groups 1–14 years had the lowest contributions to both SGLE and SGLD due to avoidable causes.

### Age- & cause-specific contributions to the sex gap in LE and LD

**Fig. 2:**
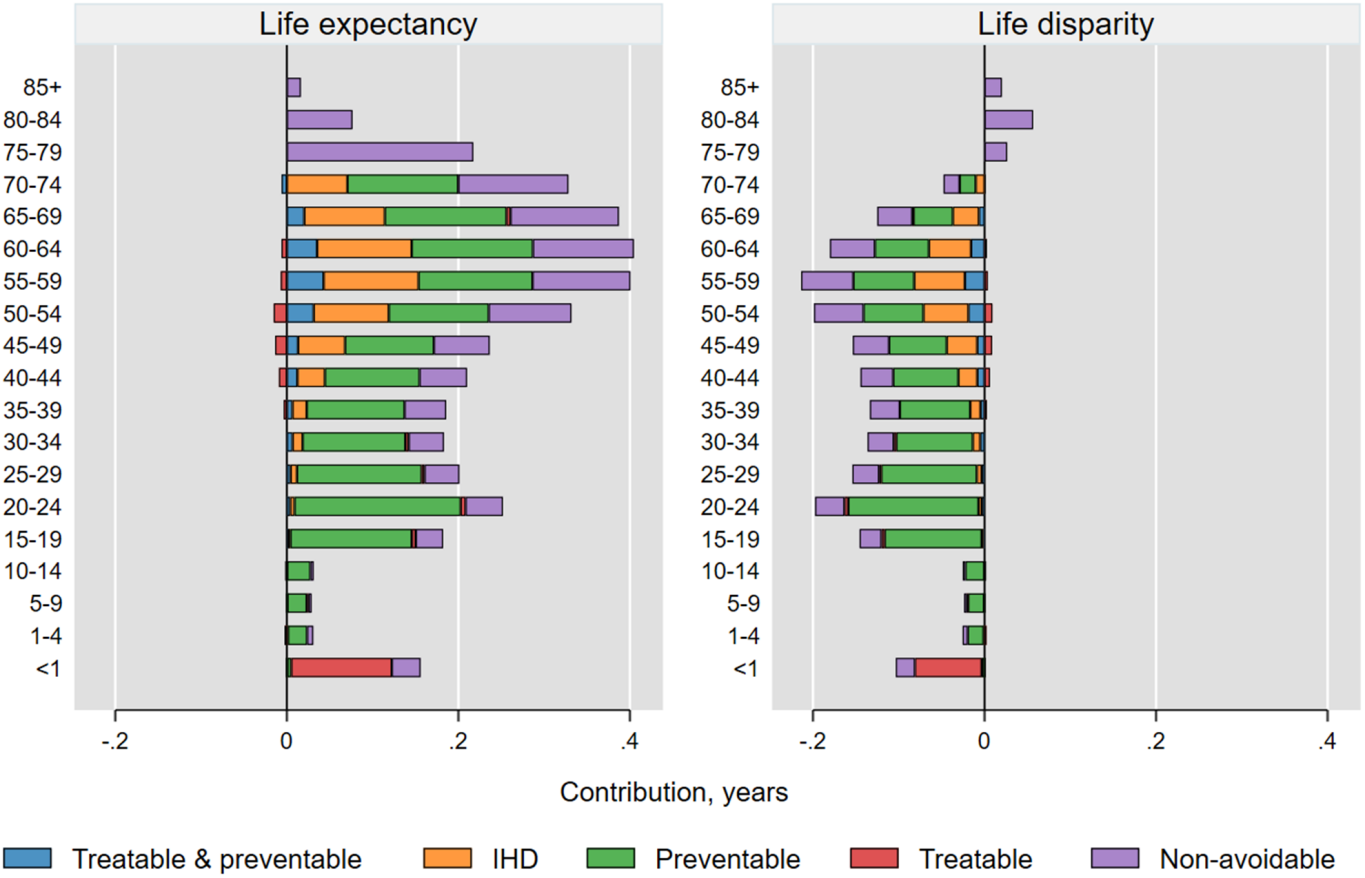
Age- and cause-specific contributions to the sex gap in life expectancy (SGLE) and lifespan disparity (SGLD) in Iran over 2015–2016 (representing females’ values minus males’ values)

As expected, deaths related to pregnancy, childbirth and perinatal period accounted for majority of total SGLE (93%) and SGLD (91%) attributable to avoidable causes in age group < 1 year (Table A1 and A2 in Additional file 1). For age groups 1–49 years, injury-related deaths were the leading contributor accounting for 75% and 67% of total SGLE and SGLD due to avoidable causes, respectively. High injury mortality rates in these age groups resulted in injury deaths alone having higher contributions (0.8 years) to the SGLD than all treatable (0.1 years), IHD (0.3 years), all treatable and preventable (0.1 years), and all non-avoidable causes (0.4 years). In people aged 50–69 years, IHD was the leading contributor to the SGLE and SGLD across avoidable causes, while in age group 70–74 years preventable cancers including lung cancer were the leading contributor. In overall, treatable causes were the leading contributor to both SGLE and SGLD in age group < 1 year, while preventable causes had the greatest contributions in all age groups from 1 to 74 years (Fig. 2).

## Discussion

This research assessed, for the first time, the contributions of avoidable causes of death, as a measure of health system performance, to the SGLE and SGLD in Iran. On average, Iranian males had 3.8 years shorter LE and 1.9 years more life years lost than Iranian females in 2015–2016. This means that compared to females, Iranian males are encountering a double burden of shorter LE and greater uncertainty at timing of death. Avoidable causes of death largely contributed to this double burden with preventable causes particularly injuries having the greatest contributions.

Larger LE and smaller LD in females compared with males observed in the current study are consistent with previous research conducted in other locations [3–5, 21]. Genetic and hormonal differences, as well as behavioral and social differences between females and males has been suggested as possible explanations [22, 23]. In addition, sex differences in health-seeking behaviors has also been suggested as a potential explanation [24]. Substantial contributions of avoidable causes of death in the present study, in particular preventable causes, confirm the important role of behavioral factors in the SGLE and SGLD. These substantial contributions of avoidable causes to the SGLE were also reported in recent studies conducted in Sweden [13] and the UK [25], even though the relative contribution of avoidable causes to the SGLE reported in the present study (67%) was larger than those reported in Sweden (47%) and the UK (54%). These general overall findings highlight the crucial role of the health systems particularly public health interventions in reducing the sex gap in longevity and life disparity.

Our results indicated that injuries had the greatest contribution to the SGLE (1.1 out of 3.8 years). In comparison, injuries had the second largest contributions accounting for 0.5 out of 3.6 years of the SGLE in the UK during 2014–2016 [25]. The greater contributions of injury deaths to the SGLE in Iran is consistent with the data from the global burden of diseases 2019 where Iran had a greater sex gap in injury mortality (72.9 per 100,000 in males vs. 24.0 per 100,000 in females) compared with the global average (75.8 per 100,000 in males vs. 35.2 per 100,000 in females) [26]. A recent study across 9 countries in Eastern Mediterranean Region also reported that Iran ranked the second in terms of the contributions of injury deaths to the SGLE and SGLD [27]. Worryingly, the burden of injury deaths is most pronounced among young economically productive age population especially those 15–34 years with injuries accounting for around 85% of total SGLE and SGLD attributable to avoidable causes among them. Riskier behavior and less adherence to law in males than females, particularly among young people, might partially explain these findings [28, 29]. Safety improvement of vehicles, roads and workplaces; public training; legislation and law improvement; and increasing the access to trauma care (pre-hospital and emergency care) are among the policies that can reduce overall injury burden and hence SGLE and SGLD in the country [30–32].

Following injury, IHD and preventable cancers including lung cancer had the highest contributions to the SGLE and SGLD among the avoidable causes. Higher rates of risk factors including unhealthy behaviors such as smoking and hazardous drinking in males than females might account for large contributions of these causes to the SGLE and SGLD [33–35]. A recent meta-analysis reported that Iranian males were 2.4 times more likely to have consumed alcohol than females which is greater than the global average (1.4-fold sex gap) [36]. Moreover, the pooled prevalence of smoking and hookah smoking in Iranian males vs. females were 10% vs. 5% and 24% vs. 13%, respectively [37]. These behavioral differences may have been driven by traditional, cultural and social norms in Iran. For example, many Iranian females are prohibited by their parents or husband to drink alcohol or smoke [38, 39].

While cross-study comparison is generally difficult due to the differences in the list of causes of avoidable death, a previous study in the UK [25] applied the same definition of avoidable causes as the present study. While the magnitudes of the SGLE were comparable (3.6 years in the UK vs. 3.8 years in Iran), avoidable causes accounted for a larger portion of the SGLE in Iran (67% vs. 54%). Of note, while treatable causes contributed to LE advantage for female in Iran, the opposite was the case in the UK. This was mainly due to larger contribution of treatable cancers (-0.34 vs. -0.17 years) and smaller contribution of pregnancy, childbirth and perinatal deaths (0.041 vs. 0.104 years) in the UK vs. Iran. Other notable differences include a higher relative contribution of cerebrovascular causes to the SGLD in Iran (4.1% vs. 2.8% of the SGLE due to avoidable causes in people aged < 75 years) and substantially larger relative contributions of alcohol– (9.0% vs. 1.2%) and drug–related (8.8% vs. 3.3%) deaths in the UK. Less pronounced contributions of alcohol–related deaths in Iran might be due to a lower consumption of alcohol considering its socio-cultural and religious contexts [36]. These factors might have also resulted in cross-country differences in death coding practices where alcohol are less likely to be recorded as an underlying cause of death in Iran, particularly considering that alcohol consumption is officially banned in the country. The substantial contribution of avoidable causes particularly preventable causes highlights the urgent need to improve intersectoral collaborations beyond healthcare system to develop coordinated public health policies targeting those at higher risk of premature death especially younger males in Iran.

Although most of the causes contributed to the LE and LD advantage for females over males, diabetes mellitus and treatable cancers contributed to the LE and LD advantage for males. Treatable cancer advantage is mainly due to breast cancer which rarely occurs among males. The higher mortality rates from diabetes for females than males in Iran might be due to higher prevalence of diabetes among females [40, 41] which is attributed to lower physical activity and higher prevalence of obesity among Iranian females than males [42–44].

### Limitations and strengths

The present study had some limitations which should be considered. First, we used the WHO mortality database to obtain data on causes of death. This source includes medically certified deaths as reported by the member countries which are known to suffer from coding errors, diagnostic inaccuracy, and underreporting. In particular, the quality of Iran’s civil registration and vital statistics is reported to be at a medium level with a completeness of 82% and usability of 67–70% [45]. Hence, potential sex differences in these errors and quality of vital registration can bias our estimates. For instance, due to social norms it might be more likely to record drug-related death as a cause of death for males than females. Second, due to the lack of insufficient trend data, we couldn’t investigate temporal changes in the SGLE/SGLD and contributions of avoidable causes to these changes. Third, the list of avoidable causes of death used in the study was developed for high income countries which might not reflect the situation in Iran. For instance, a cause of death considered as treatable in one country might not be treatable in another country due to difference in timing of the access to treatments [46]. In addition, avoidable mortality doesn’t account for the underlying prevalence of diseases and their severity as well as the influences of health policies and interventions on quality of life. Hence, it is an incomplete measure of healthcare quality and performance. Despite these limitations, our study is among first studies assessing the contributions of avoidable mortality to the SGLE in a low/middle income country. Moreover, there is limited evidence on contributions of avoidable causes to the SGLD even in high income countries.

## Conclusion

We found that more than two third of shorter LE and greater life years lost at death among males compared with females in Iran were attributable to the avoidable, particularly preventable, causes of death. In order to narrow these sex gaps in LE/LD, public health policies and interventions should especially target injuries in young males as well as heart diseases and preventable cancers in middle aged males. Moreover, improvements in maternity care should also be given a high priority. Toward a better targeted personalized healthcare, future studies should investigate the contributions of avoidable causes to socioeconomic and geographical gaps in LE and LD in the country.

## Electronic supplementary material

Below is the link to the electronic supplementary material.

**Additional file 1: Table A1.** Age- and cause-specific contributions to sex gap in life expectancy in Iran. **Table A2.** Age- and cause-specific contributions to sex gap in life disparity in Iran.

## Data Availability

All data analysed in this study is shared publicly at the WHO website.

## Abbreviations

LE: Life expectancy
LD: Life disparity
SGLE: Sex gap in life expectancy
SGLD: Sex gap in life disparity
WHO: World Health Organization
